# Discovery of a Dicer-Independent, Cell-Type Dependent Alternate Targeting Sequence Generator: Implications in Gene Silencing & Pooled RNAi Screens

**DOI:** 10.1371/journal.pone.0100676

**Published:** 2014-07-02

**Authors:** Bhavneet Bhinder, David Shum, Mu Li, Glorymar Ibáñez, Alexander V. Vlassov, Susan Magdaleno, Hakim Djaballah

**Affiliations:** 1 HTS Core Facility, Memorial Sloan Kettering Cancer Center, New York, New York, United States of America; 2 Thermo Fisher Scientific, Austin, Texas, United States of America; Beckman Research Institute of the City of Hope, United States of America

## Abstract

There is an acceptance that plasmid-based delivery of interfering RNA always generates the intended targeting sequences in cells, making it as specific as its synthetic counterpart. However, recent studies have reported on cellular inefficiencies of the former, especially in light of emerging gene discordance at inter-screen level and across formats. Focusing primarily on the TRC plasmid-based shRNA hairpins, we reasoned that alleged specificities were perhaps compromised due to altered processing; resulting in a multitude of random interfering sequences. For this purpose, we opted to study the processing of hairpin TRCN#40273 targeting *CTTN*; which showed activity in a miRNA-21 gain-of-function shRNA screen, but inactive when used as an siRNA duplex. Using a previously described walk-through method, we identified 36 theoretical cleavage variants resulting in 78 potential siRNA duplexes targeting 53 genes. We synthesized and tested all of them. Surprisingly, six duplexes targeting *ASH1L*, *DROSHA*, *GNG7*, *PRKCH*, *THEM4*, and *WDR92* scored as active. QRT-PCR analysis on hairpin transduced reporter cells confirmed knockdown of all six genes, besides *CTTN*; revealing a surprising 7 gene-signature perturbation by this one single hairpin. We expanded our qRT-PCR studies to 26 additional cell lines and observed unique knockdown profiles associated with each cell line tested; even for those lacking functional *DICER1* gene suggesting no obvious dependence on dicer for shRNA hairpin processing; contrary to published models. Taken together, we report on a novel dicer independent, cell-type dependent mechanism for non-specific RNAi gene silencing we coin **A**lternate **T**argeting **S**equence **G**enerator (ATSG). In summary, ATSG adds another dimension to the already complex interpretation of RNAi screening data, and provides for the first time strong evidence in support of arrayed screening, and questions the scientific merits of performing pooled RNAi screens, where deconvolution of up to genome-scale pools is indispensable for target identification.

## Introduction

RNA interference (RNAi) screening emerged as an important investigational tool in the post-genomic era, enabling scientists to study gene function and to validate targets rapidly [1]–[2]. In the past decade, RNAi screening has been most widely used to study genetic variabilities associated with cancer cells, and with a potential to identify novel targets and elucidate disease pathways for targeted therapy [1]–[3]. Scientists can now perform RNAi screens using a focused set of genes and up to a complete mammalian genome, in arrayed as well as pooled formats [1], [4]. Advances in technology have extended the capabilities to conduct RNAi screens in not only hard to transfect cells but have also enabled the whole organism screens, as has been recently reported in mice [5]–[6]. Indeed the technological developments have opened multiple avenues to explore the RNAi screening platform in a broader spectrum. Albeit such progress, the data outputs from RNAi screens have repeatedly failed to reproduce when tested independently [4], [6]–[11]. The upheaval of examples with regards to data discordance has, more than ever, ascertained the need to diligently address the current pitfalls of RNAi data outputs. This process would require in-depth understanding of the underlying causes and importantly, an effort to expand our knowledge of currently unknown facets pertaining to non-specific gene silencing.

Sequence based off-target effects (OTEs) are believed to be the main culprits of non-specific gene silencing. As early as 2003, Jackson and co-workers reported on random interference when using short interfering RNA (siRNA) duplexes. Their gene expression profiles showed down-regulation of non-specific genes, which bore partial sequence complementarity with the duplexes [12]. Several investigations on existence of OTEs followed thereafter [13]–[16]. OTEs displayed a tendency of being enriched in top scoring hits obtained from RNAi screens, creating a high risk of misinformation [17]–[18]. Efforts were made to address these issues and reduce the occurrence of OTEs, especially in siRNA duplexes [19]–[23]. As an example, commercially available siRNA libraries now harbor chemical modifications to increase target specificity and to allow for a guide strand bias [19]. Computational methods have also been proposed to predict OTEs in RNAi screening data outputs [21]–[23]. However, the knowledge of non-specific gene silencing has been fairly restricted to OTEs and its two key drivers that of seed match or partial guide strand match with a transcript; all endeavors have been catered towards mitigating random silencing from this perspective.

Noticeably, although both the leading RNAi technologies, siRNA duplexes and short hairpin RNAs (shRNAs), are vulnerable to OTEs, most of the work appeared to be siRNA centric. [12]–[21]. Additional sources of non-specific gene silencing that might be exclusive to shRNA hairpins remained poorly understood. siRNAs are introduced into the cells as duplexes with a pre-defined guide and passenger strand. Hence, the sequence of a guide strand inside the cell becomes a known entity. This characteristic of siRNA duplexes is a major point of difference in comparison to the shRNA hairpins, which are introduced into the cell as plasmid vectors packaged inside pseudotyped lentiviral particles. The shRNA hairpins, predominantly designed either under The RNAi Consortium (TRC) guidelines [24] or with a miR-30 backbone [25], are dependent on the host’s intracellular machinery for efficient maturation to produce interfering sequences. shRNA hairpins are believed to mimic microRNA (miRNA) biogenesis, and therefore likely to use dicer for their processing [26]; although such involvement of dicer for all hairpins has never been validated. Nonetheless, the emphasis is on a precise and efficient intracellular cleavage of hairpins at specific sites, as predicted theoretically and widely accepted to hold true. A recent work by Gu and co-workers provide the first line of contrary evidence showcasing heterogeneous intracellular cleavage of such a miR-30 based shRNA hairpin, resulting in the generation of several cleavage variants found to be dicer dependent [27]. The question emerges as to whether these variants of alternate processing would be degraded inside the cell or would these lead to silencing of non-target genes?

This aspect of alternate processing presents an unexplored and novel outlook on non-specific gene silencing originating from shRNA screens, and perhaps a reason behind data discordance and poor gene target confirmation rates. It is commonly believed that the issue of poor data reproducibility most likely originates from difference in experimental set-up, types of assays, and difference in data analysis practices [7]. In our previous work motivated towards understanding RNAi data reproducibility, we had reported on a comparative analysis amongst two genome-wide RNAi screens, both of which were performed to identify modulators of miRNA biogenesis. Based on the previous explanations for poor reproducibility, we thought that the best case scenario would be to control for all such environmental variabilities by comparing two screens conducted in the same laboratory, by the same personal, using the same assay, and readouts, and the data to be analyzed by the same methodology; we hoped to observe an excellent overlap amongst the hit lists thus obtained. The only difference in this comparative analysis was the choice of technology, one screen was siRNA based while the other was shRNA based; execution of the siRNA screen had lead to nomination of 1,274 gene candidates and the shRNA had lead to nomination of 497 gene candidates [11], [28]–[29]. The benchmark for these screens was the identification of known genes from the miRNA biogenesis pathway, and it was a matter of concern when *DROSHA* was the only known modulator identified in the shRNA screen. To our surprise, the inter-screen overlap was also dismal, with only 29 gene candidates scored as common amongst the two screens; this observation provided contradictory evidence to the previous explanations towards poor data reproducibility.

We were keen to understand the causal factors driving such discrepancies in nominated hits amongst our screens and believed that perhaps the true explanation lay in the only point of difference, which was the choice of technology. In a recent report, Ramji and co-workers had made a similar observation pertaining to non-specific outcomes from plasmid based hairpins, contrary to the specific silencing conferred by the siRNA counterpart [30]. So when we compared the silencing sequences from the two screening libraries, we were perplexed to observe a contrasting phenotypic outcome conferred by identical guide sequences [11]. Concentrating on the TRC plasmid-based shRNA hairpins, we decided to theoretically explore the aspect of altered cleavage as a likely factor leading to alternate hits and ultimately such a dissimilar hit list. We designed a walk-through study mimicking all possible 19 nucleotide (nt) long variants, beyond the theoretical site of cleavage, and a searched for matches with human genome, obtaining a theoretical list of random targets; this list also comprised of some of the known modulators of miRNA biogenesis pathway. We postulated a hypothesis that the alternate hairpin processing was perhaps the lead cause of a discrepant hits nominated in the shRNA screen [11].

To validate the *in silico* driven walk-through hypothesis, this study was designed with an aim to provide the first ever experimental evidence towards an alternate hairpin processing pathway and its implication on RNAi data outputs. For this purpose, we selected *CTTN* gene targeting hairpin, TRCN#40273, which had scored active in the shRNA screen, while its siRNA counterpart had exhibited no activity. The walk-through generated 36 cleavage variants, which we termed as alternate targeting sequences (ATS). The ATSs were matched against the human genome; 78 siRNA duplexes targeting 53 genes were identified and custom designed for testing in an image-based biosensor assay measuring gain in Enhanced Green Fluorescent Protein (EGFP) signal intensity [29]. Six of these siRNA duplexes targeting alternate genes *ASH1L*, *DROSHA*, *GNG7*, *PRKCH*, *THEM4*, and *WDR92* scored positive. qRT-PCR analysis of TRCN#40273 transduced reporter cells further confirmed the intracellular knockdown (KD) of target *CTTN* as well as all of the six alternate targets; providing a unique 7 gene-signature associated with TRCN#40273. We went a step further to perform similar qRT-PCR analysis on 20 additional cell lines and observed cell-type specific nature of KDs conferred by this gene-signature. Surprisingly, our results using 6 *DICER1* mutated and wild-type cell lines also show that ATSG is observed irrespective of the *DICER1* status in the cells. Altogether, our results shed light on a previously unknown phenomenon of cell-type dependent non-specific gene silencing in context of shRNA hairpin screens, a novel mechanism we termed as alternate targeting sequence generator (ATSG), and is found to be dicer-independent.

## Materials and Methods

### shRNA used in walk-through study

The shRNA hairpin selected for the walk-through studies was TRCN#40273, which targets the gene *CTTN*. This hairpin is a part of TRC1 collection designed by the Broad Institute (Sigma-Aldrich, St Louis, MO). This shRNA had an identical guide sequence match with the Silencer Select siRNA s4665 (Thermo Fisher Scientific, Waltham, MA). The sequence of TRCN#40273 is:

CCGGCGGCAAATACGGTATCGACAACTCGAGTTGTCGATACCGTATTTGCCGTTTTTG. The sequence of s4665 is: TTGTCGATACCGTATTTGCCG.

### Cell culture and materials

The miR-21 EGFP based biosensor (HeLaS3 miR-21 EGFPB) harboring a reporter for miRNA activity was generated as previously described. In brief, HeLaS3 cells were transfected with pcDNA/TO/EGFPmiR21 (Addgene, Cambridge, MA) using Lipofectamine 2000 transfection reagent and Zeocin-resistant cells were harvested for storage of cell stocks at −170°C. A549, 451Lu, A375, H460, H838, H1435, H2030, HCC1954, HEK293, HeLa, HeLaS3, MDA-MB-231, RPE, SK-MEL-5, SK-MEL-28, SK-MEL-94, SK-MEL-100, SK-MES-1 cell lines were purchased from American Type Culture Collection (ATCC, Manassas, VA). JIMT-1 cell line was purchased from AddexBio (San Diego, CA). LK-2 cell line was purchased from RIKEN BioResource Center (Japan). The isogenic *DICER1* knockout pairs in DLD1, HCT116, and RKO cell lines were purchased from Horizon Discovery (Cambridge, United Kingdom). All cell lines were cultured at 37°C with 5% CO_2_-95% air; in addition, supplies were from Thermo Fisher Scientific (Waltham, MA) and Sigma-Aldrich (St Louis, MO).

### Liquid dispensing and automation

Several liquid dispensing devices were used throughout this study. siRNA walk-through duplexes and shRNA lentiviral particles were transferred using a 384 stainless steel head with disposable low-volume polypropylene tips on a PP-384-M Personal Pipettor (Apricot Designs, Monrovia, CA). The addition of cell suspensions and growth media was performed using the Multidrop 384 (Thermo Fisher Scientific). Cell fixation and staining was performed using the ELx405 automated washer (BioTek, Winooski, VT).

### Walk-through siRNA studies

The 78 siRNA sequence variants (Thermo Fisher Scientific) generated from the walk-through were tested for activity using the HeLaS3 miR-21 EGFPB. Stock solutions of the siRNA duplexes were made in nuclease-free water at 100 µM concentration and diluted to 1 µM in which 5 µL was transferred into the 384-well microtiter plates to achieve a final concentration of 50 nM. For internal reference, each assay plate contained Silencer Select Negative Control #1 siRNA (catalog #4390843) as background control at a final concentration of 50 nM. Next, 10 µL/well Opti-MEM media was added followed by 15 µL/well Lipofectamine RNAi Max transfection solution at a final concentration of 0.1 µL/well and incubated at room temperature for 20 min to promote siRNA-transfection reagent complex formation. Next, cell suspensions, 500 cells per well, were dispensed into the assay plates in 50 µL growth media. At day 6 post-transfection, cells were fixed and stained followed by imaging on the IN Cell Analyzer 3000 (INCA3000, GE Healthcare, Piscataway, NJ) for EGFP fluorescence intensity and Hoechst-stained nuclei. For the pooled studies, the nominated active siRNA duplexes were combined into a single tube and diluted to 1 µM for the assay. Similarly, inactive siRNA duplexes and all siRNA duplex variants were combined and subsequently diluted to 1 µM concentration. The assay was run in HeLaS3 miR-21 EGFPB and at day 6 post-transfection; cells were fixed and stained followed by imaging on the INCA3000 as previously described.

### Image acquisition

Images were acquired on the INCA3000, an automated laser confocal microscope using the following wavelengths: 364 nm excitation/450 nm emission in the blue channel for Hoechst-stained nuclei and 488 nm excitation/535 nm emission in the green channel for EGFP signal with an exposure time of 1.5 msec. For the walk-through studies, nine images per well were collected using a 40x magnifying objective covering 90% of the well. Images were analyzed using the Raven 1.0 software’s built-in object intensity analysis module to assess green fluorescence intensity per well and count number of Hoechst-stained nuclei.

### Walk-through assessment of shRNA alternate processing

For the shRNA studies, cell suspensions at 500 cells per well were first dispensed into 384-well microtiter plates in 45 µL media. After overnight incubation, media was aspirated from the assay plates and replaced with media plus 8 µg/mL of polybrene. TRCN#40273 targeting *CTTN* (Sigma-Aldrich) was thawed from storage at room temperature and diluted in plain DMEM; after which 1 µL was transferred into the assay plates to achieve a final MOI of 4. The assay plates were briefly centrifuged for 8 min at a speed of 1,300 rpm. At day 3 post-transduction, media was aspirated and 45 µL of media containing puromycin was added to the cells and further incubated to complete selection. At day 7 post-selection, cells were harvested using the MagMAX-96 Total RNA Isolation Kit (Thermo Fisher Scientific) following manufacturer’s specifications. As internal reference, we included non-transduced cells with no puromycin, Non-Target shRNA #1 (Sigma-Aldrich), and TurboGFP (Sigma-Aldrich) as controls for transduction assessment.

### Gene expression analysis using quantitative real-time PCR

Total RNA extracted from shRNA transduction experiments were diluted with nuclease free water to a concentration of 2 ng/µL. For mRNA expression analysis, the total RNA was reverse transcribed using MMLV Reverse Transcriptase and buffers (Thermo Fisher Scientific, catalog #AM2044). For miRNA expression analysis, the total RNA was reverse transcribed using Multiscriptase E and buffers in TaqMan MicroRNA Reverse Transcription Kit (Thermo Fisher Scientific, catalog #4366597). Gene expression analysis was run using TaqMan Universal Gene Expression Mastermix II (Thermo Fisher Scientific) according to manufacturer’s specifications with the following primer/probe sets for targets: *ASH1L* (Hs00218516_m1), *CTTN* (Hs01124225_m1), *DICER1* (Hs00998588_g1), *DROSHA* (Hs00203008_m1), *GNG7* (Hs00192999_m1), *PRKCH* (Hs00178933_m1), *THEM4* (Hs00940011_g1), *WDR92* (Hs00399033_m1), and miR-21 (000397). As a reference control, Human Euk 18S rRNA (4352930E) was used for normalization of qRT-PCR data.

### Western blot analysis

Cells were lysed with RIPA buffer (Sigma-Aldrich) containing 1 mM phenylmethylsulfonyl fluoride and 2 mM tris(2-carboxyethyl)phosphine hydrochloride by sonication at 60 amplitude for 10 min on a water/ice mix. Cellular debris was removed by centrifugation at 13,000 rpm for 30 min. 20 µg for HCT116, and 40 µg for RKO and DLD1 samples were separated in 3–8% tris-acetate gels at 150 V for 55 min (BIORAD, Berkeley, CA) and transferred onto PVDF membrane (Millipore, Billerica, MA) at 4°C and 100 V for 2 h. Membranes were blocked with 5% non fat dry milk (BIORAD) in PBS containing 0.1% Tween-20 for 1 h at room temperature. The membranes were incubated independently with anti-*DICER1* antibody (1∶500, Abcam, Cambridge, MA) and anti-Actin (1∶400, Abcam) overnight at 4°C, followed by 1 h incubation at room temperature with horseradish peroxidase-conjugated secondary antibody (1∶5000, Cell Signaling, Danvers, MA). The images were analyzed and quantified using ImageJ.

### Statistical data analysis of the walk-through studies

For the purpose of evaluating the activity of individual siRNA duplexes in the experiment, the data values corresponding to the gain in EGFP fluorescence intensity were obtained and converted into percentage gain (% gain) relative to the Silencer Select Negative Control No. 1 calculated using the following formula:
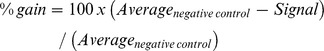



The threshold was determined at a % gain in EGFP signal to be at least 2 standard deviations above the mean of the negative control. In parallel, the cytotoxicity of individual siRNA duplexes was determined using the residual nuclei count (NUCL), also converted into percentage inhibition (% inhib) calculated using the following formula:
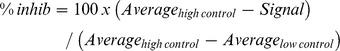
Where, the high control is Silencer Select Negative Control No. 1 and the low control is PLK1 (s449). The threshold to score for cytotoxic siRNA duplexes was set at atleast 50% kill relative to the controls. All siRNA duplexes were screened in quadruplicates and the corresponding values were averaged for calculating a single % gain and % inhib for each siRNA. The data analysis was done using Perl scripts and the graphical representation for data visualization was done using SigmaPlot (Systat Software Inc., San Jose, CA).

## Results

### Selection of *CTTN* shRNA (TRCN#40273) for walk-through study

The walk-through study was designed to investigate the existence of alternate hairpin processing pathway, beyond its theoretical site of cleavage, and to determine if these altered targeting sequences would modulate the EGFP signal intensities in a miRNA-21 gain-of-function biosensor assay. For the purpose of walk-through study, we decided to select a hairpin from the TRC1 library, based on its performance in the previously described miRNA-21 gain-of-function genome-wide screen [29]. A set of five criteria were charted out to qualify a hairpin for consideration in walk-through experiments, which were: 1) targeting sequence of the hairpin must have an identical match with that of an siRNA duplex tested under similar experimental set-up [28], 2) this targeting sequence must be active exclusively in shRNA hairpin screen, but inactive in siRNA duplex screen, 3) the hairpin, thus picked, has been independently validated to produce at least 50% knockdown of its target, 4) the target of this hairpin has no *a priori* described role in miRNA biogenesis pathway, and 5) altered processing of this hairpin would yield targeting sequences which could silence known modulators of the miRNA biogenesis, the pathway of interest in the screen.

Based on these selection criteria, we inspected a collection of hairpins that scored active in the shRNA screen and randomly picked hairpin TRCN#40273, targeting *CTTN*, to further assess its feasibility to be used in walk-through study. TRCN#40273 was scored active based on a 35% gain in EGFP signal relative to the controls used in this screen [29]. Of note, the threshold for minimal hairpin activity in the shRNA screen was set at a minimal 25% gain in EGFP signal [29]. An identical targeting sequence to TRCN#40273 was found in Silencer Select siRNA s4665; this duplex had scored inactive in the miRNA-21 gain-of-function siRNA screen. TRCN#40273 has also been independently validated by Sigma-Aldrich, in collaboration with the Broad Institute, in the A549 cell line, and was reported to produce approx. 85% reduction in mRNA levels of its target *CTTN*. Furthermore, to our knowledge *CTTN* has not been implicated to play any known role in miRNA biogenesis pathway. Interestingly, a sequence-based search of various likely cleavage variants of TRCN#40273 identified partial matches with, amongst others, a well-established modulator of miRNA biogenesis pathway, *DROSHA*. In addition, a seed sequence match assessment revealed its similarity to 2 miRNAs, miR-549 and miR-4444. Since TRCN#40273 met our qualifying criterion in entirety, we selected it to be tested experimentally for existence of alternate processing hypothesis.

### Theoretical design of the walk-through study: identifying targets of alternate processing

**Figure 1:**
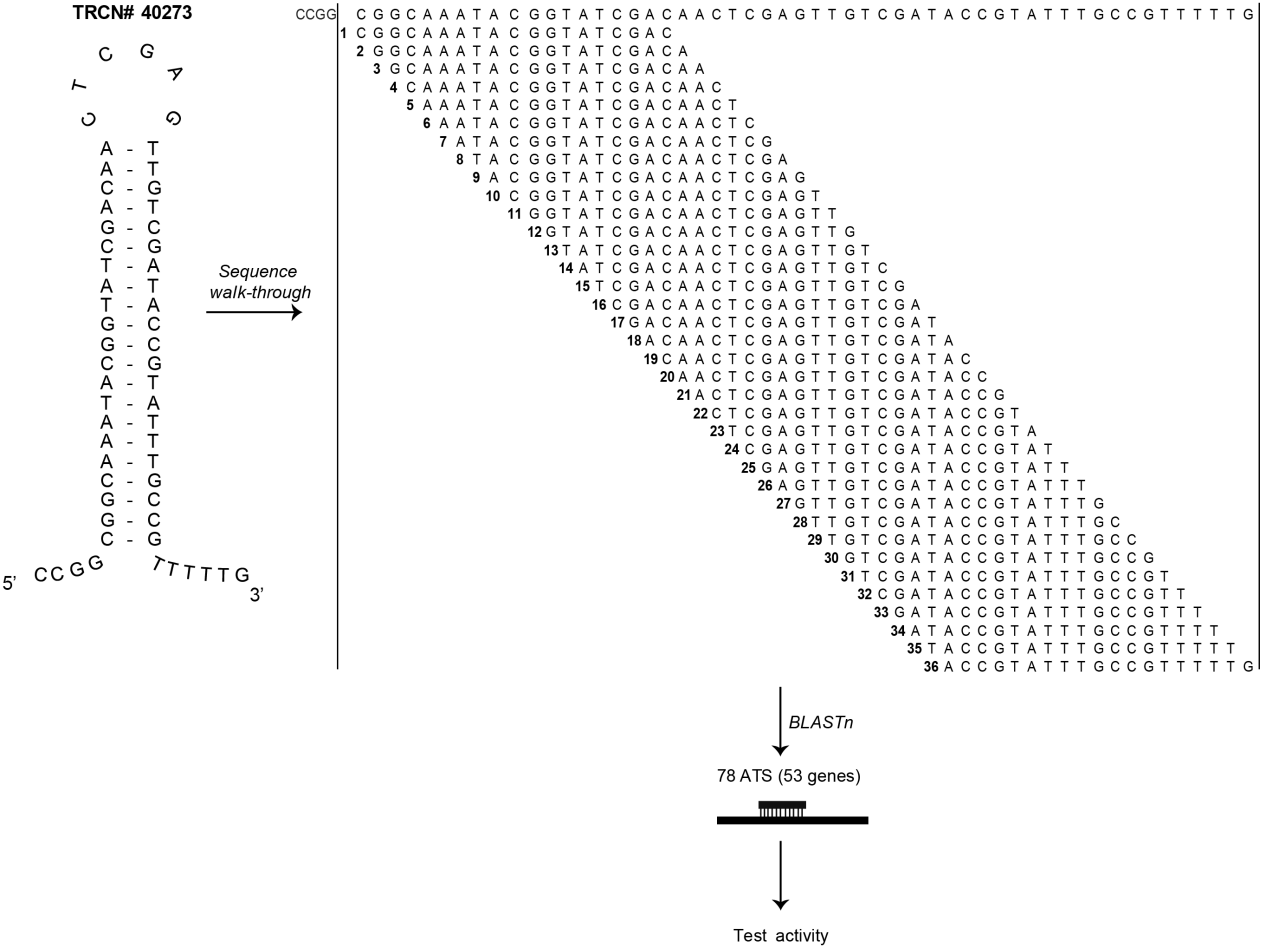
Schematics of the walk-through study using TRCN#40273. Hairpin cleavage variants identified for the entire length of the oligonucleotide with a step size of 1

The concept of walk-through has been previously described in detail [11]. Briefly, we split an shRNA oligonucleotide into all possible 19 nt long guide alternate targeting sequences (ATS) indicative of all possible sites of hairpin cleavage, starting from its 5′ end after excluding the 5′ overhangs, and incrementing by one nucleotide each time (**Fig. 1**). Following these steps, processing of a shRNA oligonucleotide would generate 36 to 37 theoretical ATS per hairpin. Since hairpin TRCN#40273 is 58 nt long, a total of 36 ATS were generated and the reverse complements of these ATS were individually searched for matches against the human mRNA databases using the Standard Nucleotide Basic Local Alignment Search Tool (BLASTn) [31]. Complete as well as partial sequence matches were considered in this analysis so as to obtain a list of all possible targets that were non-specific for the hairpin. Top blast results were inspected for degree of sequence overlaps with ATS, and those corresponding to perfect matches especially in the region of seed heptamers were given more weighting in the selection process. Finally, 78 sequences targeting 53 alternate target genes, including the actual target *CTTN*, were shortlisted for further evaluation. siRNA duplexes corresponding to these 78 ATS were custom designed to be tested in the walk-through experiments (**Fig. 1**, **Table S1**).

### 6 sequence variants of shRNA exhibit functional activity in walk-through studies

**Figure 2:**
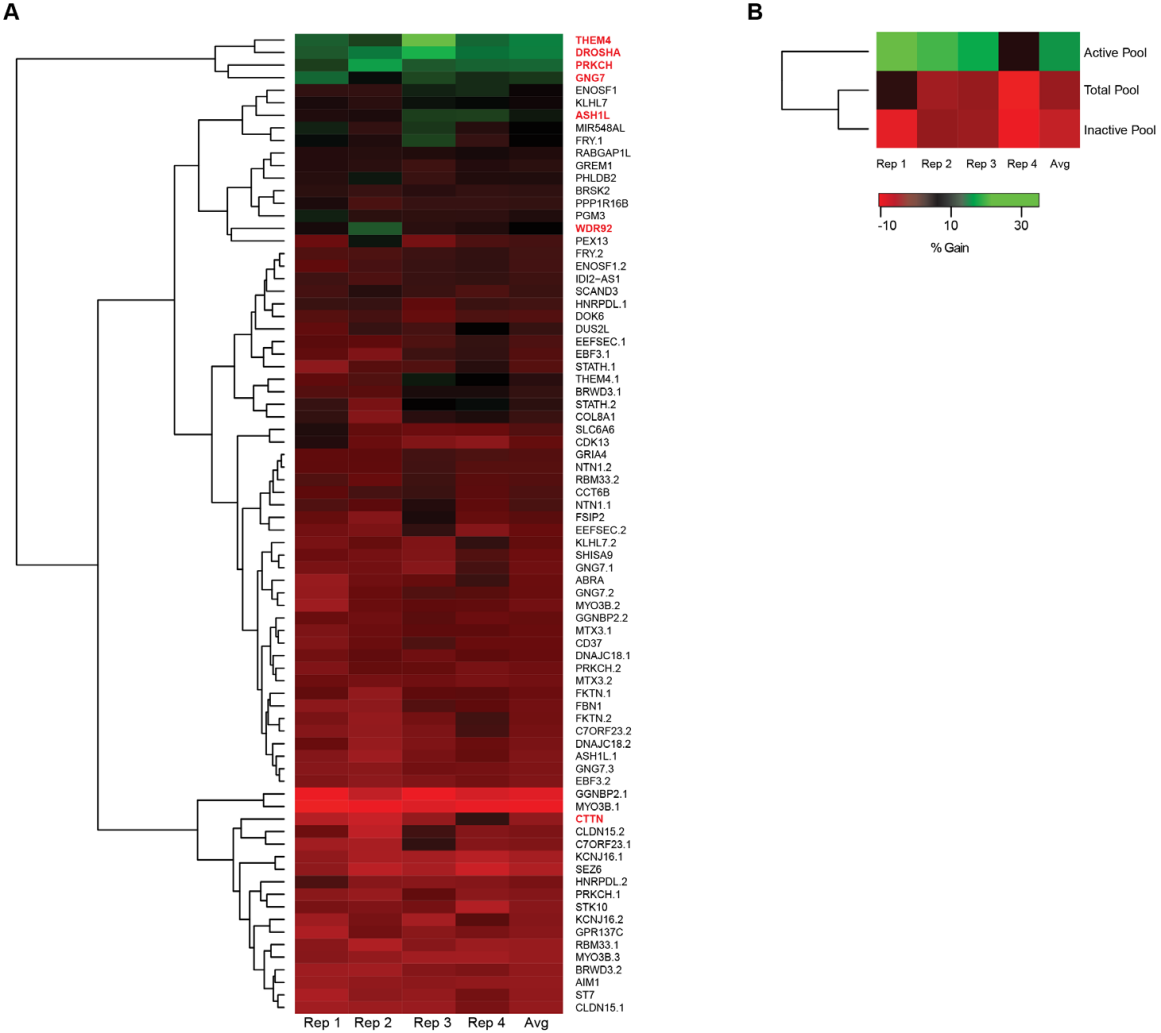
Walk-through experiment results for 78 ATS siRNA duplexes. Results of walk-through experiments measured at day 6 post transfection with synthetic siRNA duplexes using EGFPB reporter cell line. (**A**) Clustered heatmap to show % gain in EGFP signal conferred by 78 ATS siRNA duplexes, tested as singles. (**B**) Clustered heatmap to show % gain in EGFP signal conferred by siRNA duplexes segregated into three pools that of duplexes active as singles, inactive as singles or with all inclusive. Rep stands for replicate, AVG stands for average of the four replicates.

We aimed to experimentally review the activity of the ATS that might be generated intracellularly due to differential hairpin cleavage. The walk-through study was set-up to mimic the primary shRNA screen [29]; an image-based biosensor assay was conducted in HeLaS3 miR-21 EGFPB cell line utilizing the 78 ATS derived siRNA duplexes (**Fig. 1**, **Table S1**). In its first phase, individual siRNA duplexes were tested as singles. siRNA duplexes were assessed for an up-regulation of EGFP fluorescence intensity signal as well as minimal cellular toxicity, which was measured simultaneously by recording the NUCL. The siRNA duplexes were tested in quadruplicates and the resulting values were averaged for the purpose of processing. Statistical data analysis revealed that 6 siRNA duplexes targeting the genes *ASH1L, DROSHA, GNG7, PRKCH, THEM4,* and *WDR92,* conferred a gain in EGFP signal relative to the negative control (**Fig. 2A**). These siRNA duplexes produced a marginal cell loss, quantified as less than 50% inhibition in NUCL, and therefore, were scored as non-cytotoxic to the reporter cell line. Surprisingly, the siRNA duplex designed to knockdown the actual target of TRCN#40273, *CTTN*, did not show any significant up-regulation of EGFP signal, in fact its calculated % gain value was approx. −4% (**Fig. 2A**).

In the second phase of the walk-through study, we repeated the same experimental procedure but using a pooled approach. The siRNA duplexes were divided into 3 different pools based on the outcomes from the initial phase; first pool was comprised of 6 siRNA duplexes that scored active as singles during the initial phase, second pool was of those 72 siRNA duplexes that scored inactive, and the third pool contained all of the total 78 siRNA duplexes being tested (**Fig. 2B**). Quite strikingly, the active siRNA pool produced a % gain of 22%, which was the highest relative to the other two pools. A negligible 3% gain was observed in the total siRNA duplex pool, while a very poor activity of merely 0.4% gain was found in the inactive siRNA duplex pool.

### Confirmation of alternate target knockdown in the reporter cell line: introducing ATSG

**Figure 3:**
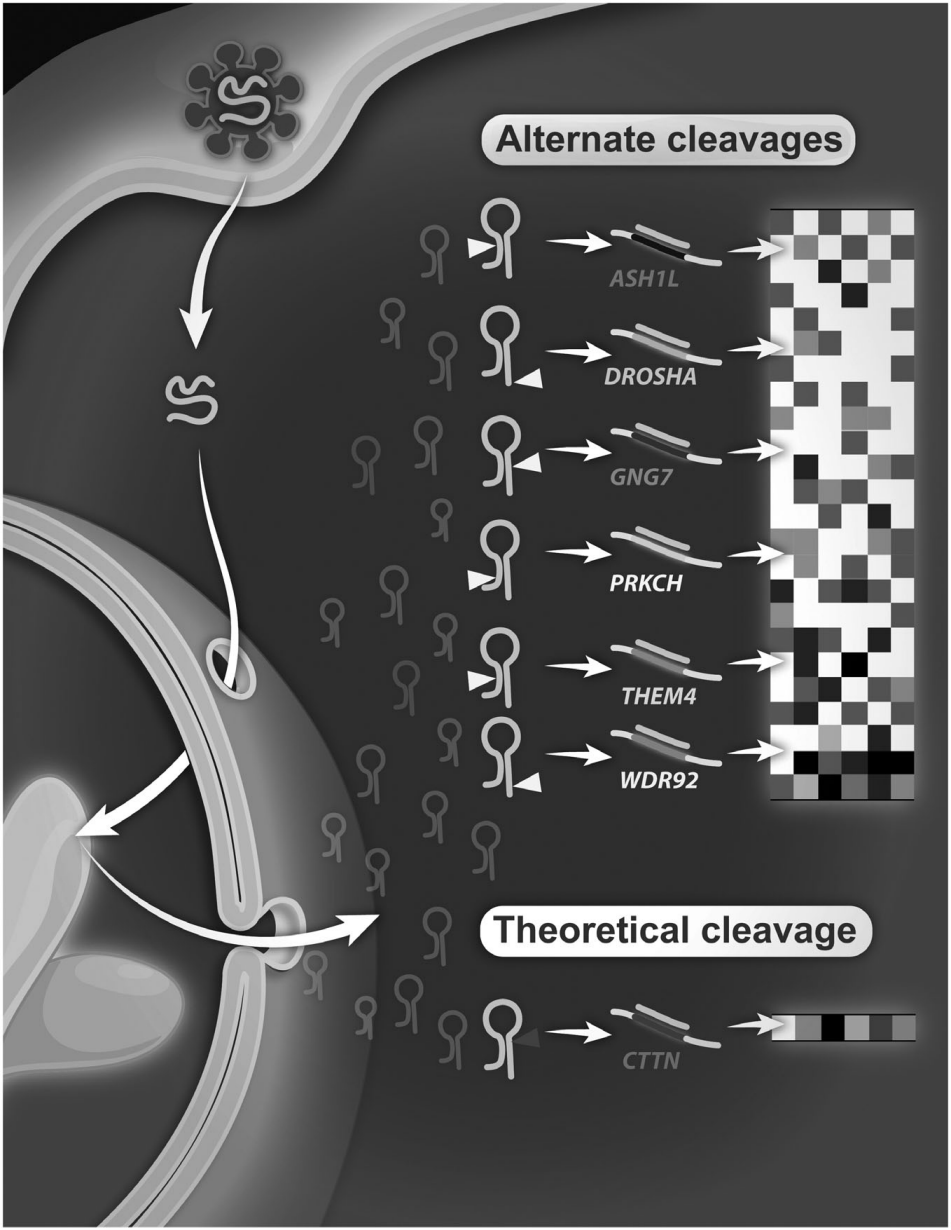
Schematics for Alternate Targeting Sequence Generator (ATSG). Hairpin inside the cell gets cleaved at its theoretical site and silences its target specifically. Inefficiencies in cleavage would lead to ATSG, generating random targeting sequencing which silence alternate targets, making it extremely difficult to comprehend the eventual phenotypic outcomes.

The results of the walk-through studies provided evidence towards existence of 6 functional ATS, by virtue of them exhibiting a gain in EGFP signal (**Fig. 3**). To determine whether the observed gene silencing was indeed conferred by these ATS, qRT-PCR experiments were set-up so as to detect reduction in mRNA levels of the corresponding alternate targets. qRT-PCR is sensitive enough to enable identification of even small changes in the mRNA levels, therefore was the technology of choice to confirm gene knockdown. For the purpose, TRCN#40273 was retested closely following the protocol standardized for the image-based biosensor assay and as implemented in the genome-wide shRNA screen [29]. HeLaS3 miR-21 EGFPB, which was the reporter cell line originally used in the gain-of-function screens, was transduced with TRCN#40273. After transduction with selection process, cells were harvested and total RNA was isolated for use in qRT-PCR reactions with the selected genes including the intended target gene, *CTTN* and 6 other alternate target genes (*ASH1L, DROSHA, GNG7, PRKCH, THEM4,* and *WDR92*).

**Figure 4:**
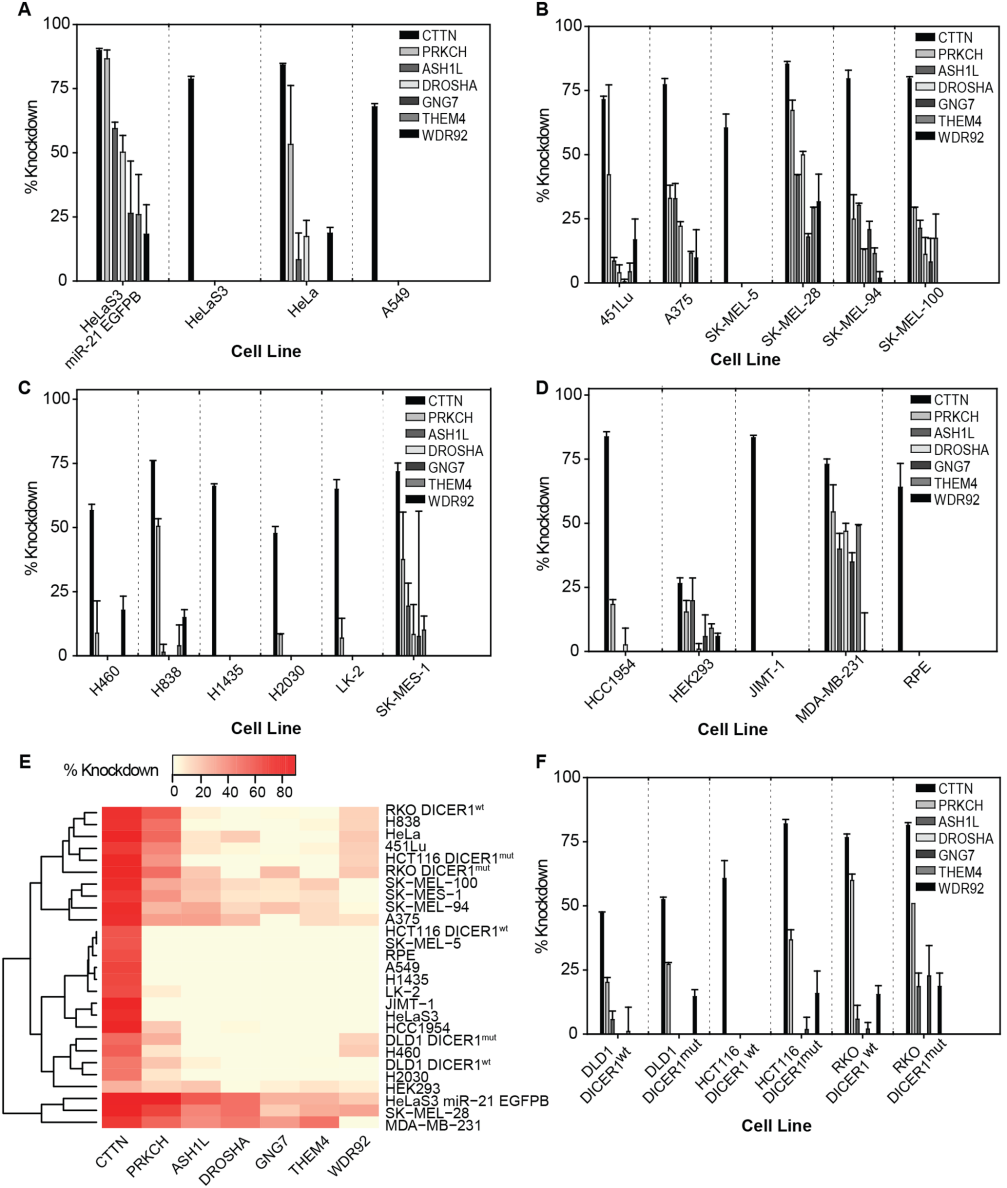
mRNA knockdown profiles for 7 gene-signature post transduction with TRCN#40273. (**A**) qRT-PCR results for panel of 4 cell lines including reporter cell line. (**B**) qRT-PCR results for panel of 6 melanoma cell lines. (**C**) qRT-PCR results for panel of 6 adenocarcinoma cell lines. (**D**) qRT-PCR results for panel of 5 cell lines derived from breast, kidney or retina. (**E**) Clustered heatmap to show mRNA knockdown levels of 7 genes across 27 distinct cell lines. (**F**) qRT-PCR results for 3 *DICER1* ^mut^ and 3 *DICER1* ^wt^ cell lines. Data in the bar graphs is expressed as average ± standard error.

Quantification of the target *CTTN* mRNA levels corresponded to an average knockdown of 90% in the HeLaS3 miR-21 EGFPB reporter cell line; indicating that the hairpin indeed perturbed its intended target (**Fig. 4A**). However, when tested as a siRNA duplex in the walk-through experiments, it had failed to confer a gain in EGFP signal. Therefore, although this hairpin down-regulated its target as expected, the target perturbation did not seem to have any correlation with gain in EGFP signal, as was observed in the shRNA screen. This motivated us to review the gene expression levels of the 6 alternate targets, as they had previously shown a phenotypic response. Surprisingly, all 6 alternate targets exhibited a heterogeneous knockdown profile ranging from 18% to 87% reduction; specifically of interest was the knockdown associated with *PRKCH* (87%), followed by *ASH1L* (59%), and *DROSHA* (50%) (**Fig. 4A**). Taken together, these findings support presence of a 7 gene-signature exhibiting modulated levels of expression when targeted by TRCN#40273 in the reporter cell line.

The walk-through studies in conjugation with the confirmation experiments provide strong experimental evidence towards existence of inefficiencies during hairpin cleavage to produce ATS, that, consequentially, silence random non-specific targets inside a cell. This phenomenon is a novel avenue for down-regulation of alternate gene targets in shRNA hairpin screens, a mechanism we termed as Alternate Targeting Sequence Generator (ATSG) (**Fig. 3**).

### Gene-signature knockdowns in 20 cell lines reveal a cell-type specific behavior of ATSG

We progressed further to assess whether the 7-gene signature, as an outcome of ATSG associated with TRCN#40273, was specific to the reporter cell line or a generic observation. The repertoire of cells was expanded to 20 different cell-types, and was reviewed under 4 panels (**Table 1**). Of note, the genes under evaluation were not expressed equivalently across all of the 20 cell lines. The parental cell line, HeLaS3, was the closest comparison to the reporter cell line utilized in the previous experiments. Hence, we first looked at expression of gene-signature in HeLaS3 and surprisingly only targeted knockdown of *CTTN* was observed (**Fig. 4A**). However, the untransformed HeLa cells did show a diverse knockdown profile, with an 84% target *CTTN* knockdown and also a 53% knockdown of the alternate target, *PRKCH*; while *ASH1L*, *DROSHA* and *WDR92* had a moderate reduction in their mRNA levels (**Fig. 4A**). Next, we evaluated the levels of knockdown in A549, the cell line of choice for independent hairpin validations performed by Sigma-Aldrich in collaboration with the Broad Institute. Here, we observed a *CTTN* knockdown of 68%, little lower than the 85% reduction as reported previously. A549 cells behaved in a manner similar to the HeLaS3 cell lines, and produced no significant down-regulation in gene expression of the 6 alternate targets (**Fig. 4A**).

**Table 1:** List of 27 total cell lines used towards confirmation of 7 gene-signature knockdowns.

Cell line	Tissue	Type
HeLaS3 miR-21 EGFPB^1^	Human cervix	Adenocarcinoma
HeLaS3	Human cervix	Adenocarcinoma
HeLa	Human cervix	Adenocarcinoma
A549^2^	Human lung	Carcinoma
451Lu	Human skin	Malignant melanoma
A375	Human skin	Malignant melanoma
SK-MEL-5	Human skin	Malignant melanoma
SK-MEL-28	Human skin	Malignant melanoma
SK-MEL-94	Human skin	Malignant melanoma
SK-MEL-100	Human skin	Malignant melanoma
H460	Human lung	Carcinoma (large cell)
H838	Human lung	Adenocarcinoma (non-small cell)
H1435	Human lung	Adenocarcinoma (non-small cell)
H2030	Human lung	Adenocarcinoma (non-small cell)
LK-2	Human lung	Squamous cell carcinoma
SK-MES-1	Human lung	Squamous cell carcinoma
HCC1954	Human breast	Ductal carcinoma
HEK293	Human embryonic kidney	Normal
JIMT-1	Human breast	Carcinoma
MDA-MB-231	Human breast	Adenocarcinoma
RPE	Human retina	Normal
***DICER1*** ** isogenic cell lines**		
DLD1 *DICER1* ^wt^	Human colon	Colorectal adenocarcinoma
DLD1 *DICER1* ^mut^	Human colon	Colorectal adenocarcinoma
HCT116 *DICER1* ^wt^	Human colon	Colorectal carcinoma
HCT116 *DICER1* ^mut^	Human colon	Colorectal carcinoma
RKO *DICER1* ^wt^	Human colon	Carcinoma
RKO *DICER1* ^mut^	Human colon	Carcinoma

1 Reporter cell line used in the screen and walk-through experiments.

2 Cell line used for TRCN#40273 validation performed by Sigma-Aldrich in collaboration with the Broad Institute.

wt; wild-type, mut; mutant.

The second panel tested was composed of 6 melanoma-derived cell lines; *CTTN* showed the highest knockdown out of all the targets tested (**Fig. 4B**). With an exception SK-MEL-5, the remaining 5 melanoma cell lines did produce a reduction in the mRNA levels of alternate targets, ranging anywhere from 4% and up to 67%. In the third panel of 6 adenocarcinoma-derived cell lines, a preferential knockdown of *CTTN* was observed across the board, and even in the case of alternate targets, the resulting knockdowns were much more conserved (**Fig. 4C**). Nonetheless, *PRKCH* yet again emerged as an interesting target in H838 (50%), and SK-MES-1 (38%) cell lines from this panel. Finally a panel of cell lines representing breast, retina, and kidney cell-types was also recruited for the analysis, and these resulted in unique signatures of knockdown for the targets (**Fig. 4D**).

Noticeably, TRCN#40273 did confer a knockdown of *CTTN* in all cell-types but at varying levels revealing that the TRC based hairpin itself might be cell-type specific in its activity towards silencing its target gene; the knockdowns varied from being as high as 90% in the reporter cell line to as low as 26% in HEK293 cell line. Also of interest was the activity of the alternate target *PRKCH,* 4 cell lines (H838, HeLa, MDA-MB-231, SK-MEL-28) out of the 20 tested, produced greater than 50% reduction in mRNA levels of this alternate target. In summary, the intended target *CTTN* was perturbed in all the cell-types tested, though at varying degrees of knockdown; while the 6 alternate targets resulted in unique and quite heterogeneous knockdown profiles which were specific to each cell-type; ranging from exclusive down-regulation of the target gene and up to down-regulation of the entire 7 gene-signature (**Fig. 4E**). The gene-signature knockdowns in 20 cell lines provide support to the fact that ATSG is most likely be a phenomenon dependent on the cell-type utilized in silencing.

### *DICER1* mutated cell lines reveal no link between *DICER1* expression and ATSG

It is commonly believed that *DICER1* might be required for intracellular cleavage shRNA hairpins [26]. Therefore, we decided to study the relationship between *DICER1* expression and ATSG mediated alternate targeting. Typically, using *DICER1* isogenic knockout cell lines would be ideal models of testing. Since, true *DICER1* knockouts are nonviable; we had to use hypomorphic mutants (*DICER1^mut^*) that were generated by disrupting exon5 of *DICER1* gene [32]. For the purpose of the experiment, we obtained three *DICER1^mut^* and three wild-type (*DICER1^wt^*) cell lines (**Table 1**), and followed through the same experimental steps of transduction, total RNA extraction and running a qRT-PCR analysis. Gene expression analysis revealed unique profiles associated with the gene-signature; no clear discriminator was found amongst the *DICER1* mutants and wild-types (**Fig. 4E**). As an example, RKO cell line produced a comparable *CTTN* target knockdown in *DICER1^wt^* (77%) and *DICER1^mut^* (81%) genotypes. In addition, a high knockdown of alternate target *PRKCH* in *DICER1^wt^* (60%) and *DICER1^mut^* (51%) was also observed (**Figs. 4E&F**). Overall, the data acquired from these analyses, provides no clear indication towards a direct association between *DICER1* expression in the cell and gene silencing produced by this hairpin, inclusive of both the intended target as well as alternate targets resulting from ATSG.

**Figure 5:**
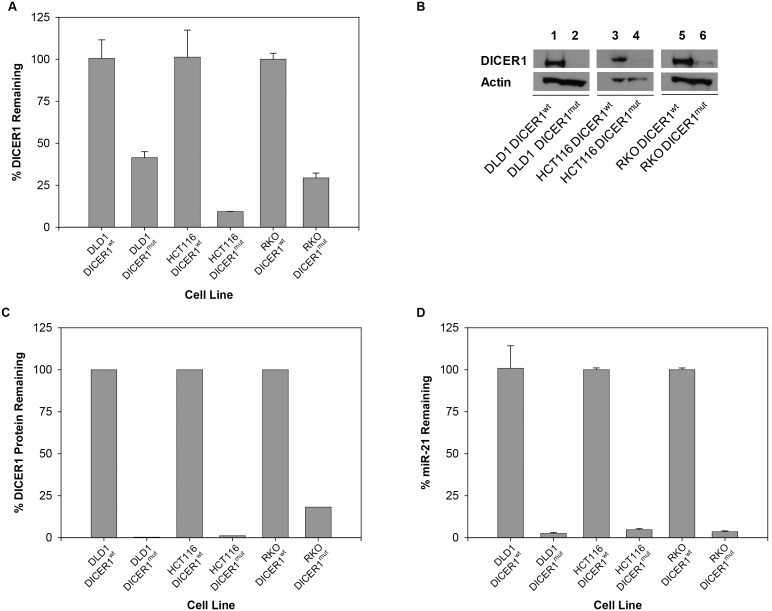
*DICER1* depleted functional reporter & protein expression. (**A**) qRT-PCR analysis to show expression levels of *DICER1* mRNA in mutant and wild-type cell lines. (**B**) Western blot analysis to show intracellular *DICER1* protein levels in mutant and wild-type cell lines. (**C**) Bar graph showing results of western blot analysis, quantified as *DICER1* protein expression relative to expression of β-actin (ImageJ). (**D**) qRT-PCR expression studies with miR-21 as reporter of *DICER1* activity.

These findings were not in agreement with previously published data with regards to dicer involvement in hairpin processing. Therefore, to support our results, we undertook a series of experiments to validate *DICER1* status by measuring the gene expression, intracellular protein levels and functionally of *DICER1* in the cell lines used. First, gene expression levels were assessed using qRT-PCR with probes targeting exon5 of *DICER1*. An overall reduction in *DICER1* mRNA levels was observed in *DICER1^mut^* relative to *DICER1^wt^* (**Fig. 5A**). Second, *DICER1* protein levels were measured using a western blot analysis; *DICER1^mut^* had almost complete protein depletion (**Figs. 5B&C**), most likely due to truncated exon5 that codes for an essential N-terminal helicase domain of this protein [32]. Finally, the functionality of *DICER1* inside the cells was determined based on its essential role in processing of many miRNAs into their mature forms [33]–[34]. Here, we selected one such miRNA, miR-21 for evaluation, and performed qRT-PCR analysis to measure amounts of mature miR-21 transcripts in the cell; *DICER1^mut^* had greater than 75% loss of miR-21 in comparison to *DICER1^wt^* (**Fig. 5D**). Taken together, the results confirm that the hypomorphic mutant cell lines indeed have depleted levels of *DICER1* mRNA and protein levels, in addition to an almost complete lack of its functionality.

## Discussion

We recently conducted a comparative analysis between siRNA duplex and shRNA hairpin based identical genome-wide screens; surprisingly, a dismal overlap of only 29 genes was observed [11]. We undertook a distinct approach to understand such discordance amongst nominated hits by reviewing differences at the level of targeting sequences. Focusing mainly on plasmid-based hairpins, we hypothesized that inefficiencies in their cleavage might be the likely culprits of random interference. In this report, we provide first ever-experimental evidence towards our hypothesis and existence of a novel mechanism, coined ATSG, driving non-specific gene silencing by plasmid-based shRNA hairpins (**Fig. 3**). We designed and implemented a walk-through study to identify 36 possible cleavage variants of shRNA hairpin, TRCN#40273 targeting gene *CTTN*. *In silico* sequence matches with the human genome enabled us to obtain 78 ATS targeting 53 unique genes to be tested in the walk-through experiment; siRNA duplexes against six genes (*ASH1L, DROSHA, GNG7, PRKCH, THEM4,* and *WDR92*) conferred a gain in EGFP signal. qRT-PCR allows for identification of even small changes in mRNA levels and was therefore used to confirm this observation in transduced reporter cell line. The results indeed validated the down-regulation of these 6 transcripts, along with the target *CTTN*. Finally, a 7 gene-signature perturbed by this hairpin was identified. When tested in 20 additional cell lines, unique KD profiles were found to be associated with each individual cell-type. Briefly, our experiments strongly support existence of ATSG as a previously unknown route for silencing random gene targets in plasmid-based hairpin screens, adding another layer of complexity in interpreting results from such screens conducted on high-throughput platforms.

It is a widely held belief that plasmid-based shRNA hairpins get processed inside the cell accurately so as to enable a target specific KD. The first wave of contradiction emerged from the observations made by Gu and co-workers [27]. They provided northern blot analyses revealing multiple sequence variants produced from miR-30 based hairpins in HEK293 cells, resulting from imprecise hairpin cleavage by dicer [27]. However, they did not proceed to show if these variants were functionally active. We took this as an opportunity to explore in detail the likelihood of alternate cleavage events in TRC designed hairpins and to assess their overall impact on RNAi data outputs. We approached the issue with a different perspective, by theoretically predicting all cleavage variants *in silico* first, and then going a step further to experimentally test these for functional activity. Based on the results from two previously described RNAi screens [28]–[29], we collected all those duplexes which possess identical targeting sequences amongst the siRNA and shRNA screening libraries; followed by filtering for only those that produced dissimilar outcomes [11]. Plasmid-based hairpin TRCN#40273 was randomly selected from this filtered pool for testing. Our hypothesis was based on the assumption that a hairpin would generate all possible 19 nt long cleavage variants inside a cell. Therefore, our starting set comprised of 36 cleavage variants derived from the 58 nt long hairpin, TRCN#40273 and each one was to be evaluated for any non-specific phenotypic response.

The BLASTn search conducted using the cleavage variants had resulted in 53 unique alternate targets of 78 ATS, each with a potential to be randomly silenced inside the cell. This list of alternate targets was obtained based on partial sequence complementarity, and the matches in most cases were imperfect ranging from 10 nt and up to 16 nt. Studies in the past had suggested that siRNA duplexes not only tend to recognize and silence their targets but also the transcripts with which they had partial sequence complementarity, sometimes of significantly small proportion. Jackson and co-workers had provided gene expression profiles to show KD of not only the target *MAPK14*, but also nine other genes that bore a minimal 8 nt similarity with the siRNA duplex [12]. Scacheri and co-workers had made similar observations, where they report protein level reduction in target MEN1 as well as two unrelated targets, p53 and p21 [16]. Furthermore, the importance of the seed region has long been emphasized in determining RNAi specificities [35]; 43 ATS in our selected list also had a perfect seed region match with their proposed targets (**Table S1**). We incorporated these features in our selection process to shortlist best representatives to be tested in walk-through studies. Finally, 6 alternate targets were found to have functional activity in walk-through study and their KD was also confirmed using gene expression analysis in the reporter cells. Our selection process taken together with the experimental results ascertained that imperfect matches with alternate targets render phenotypic outcomes; similar to what was previously reported for OTEs in siRNA duplexes, more so are manifold due multiple possible ATS per hairpin.

The shRNA hairpin libraries, either TRC or miR-30 based, have been used to perform targeted KDs in a wide variety of cell lines [4]; clearly the efficacy of a hairpin is believed to be universal. However, validation data that would confirm or challenge identical hairpin performance across different cell-types has been rarely reported [36]. Previously, in a shRNA hairpin based lethality screen, we had found that *PLK1*, an essential modulator of cellular survival and a benchmark hit, displayed unexpectedly dismal performance in HeLa cells, even when 20 out of the 23 total *PLK1* hairpins in the library were independently validated in MCF7 cells [37]. To explain this observation, we had postulated that *PLK1* hairpins perhaps underwent differential processing depending on the cell-type used. In this report, the hairpin, TRCN#40273 selected for walk-through experiments was previously validated by Sigma-Aldrich in collaboration with the Broad Institute; independent qRT-PCR experiments showed reduction in its target *CTTN* mRNA levels of approx. 85%. However, this validation was conducted in only one cell line, A549. Our findings from the gene expression analysis for target *CTTN* in a total of 27 cell lines revealed a degree of variability in the KD profiles per cell line ranging from 90% in the HeLaS3 miR-21 EGFPB reporter to 26% in HEK293 (**Figs. 4A–E**). Interestingly, the remaining 6 alternate targets exhibited even higher heterogeneity in reduction of their mRNA levels. Few cell lines, noticeably A549, rendered a target specific KD of only *CTTN* while few others appeared to produce down-regulation of the entire 7 gene-signature. Noticeable, alternate target *PRKCH* produced KDs of greater than 50% in H838, HeLa, MDA-MB-231, and SK-MEL-28. Of note, optimal transduction conditions were used for each cell line. No specific silencing pattern was observed upon examining the cell-types grouped by their origin, and KDs were deemed to be unique across the board. Hence, our validations experiments in battery of cell lines, for the first time, reveal cell dependent variations in amounts of perturbation conferred by a plasmid-based hairpin in: 1) its intended target (contrary to the generic assumption), and, 2) alternate targets. This also establishes ATSG as a cell-type specific mechanism.

Amongst the cell-types tested, we had also included 6 *DICER1* ^mut or wt^ cell lines so as to assess the influence of dicer expression on ATSG. Dicer is an RNase III type endonuclease with a key role in miRNA biogenesis pathway [33]–[34]. shRNA hairpin mediated interferences are believed to mimic miRNA-like biogenesis pathways, and are therefore dependent on dicer for their cleavage to produce siRNA like duplexes [38]. However, a dicer independent non-canonical RNAi pathway does exist; some miRNAs have been shown to seek Ago mediated cleavage instead, for their maturation [39]–[40]. It has been suggested that cleavage of shRNA hairpins might also be dicer independent most likely due to differences in the length of their stem or loop [38], [41]. The actual mechanism and factors triggering the cleavage of a hairpin through dicer independent routes is still not very clear. Since *DICER1* isogenic knockouts are not viable, we had used hypomorphic *DICER1* mutants in our analysis. Nonetheless, we validated the mutant cell lines for depletions in their levels of dicer and its activity. We observed KDs in intended as well as alternate targets, irrespective of whether the cell lines expressed dicer at normal levels or were dicer depleted. Therefore, no conclusive relationship between dicer expression and production of functional ATS was found, and perhaps the latter could be inferred as a dicer-independent phenomenon.

We have demonstrated the mechanism of ATSG in a single hairpin, silencing 6 random genes in addition to its target. Several other theoretical examples showing cleavage variants of distinct hairpins from these screens and their proposed alternate targets have also been reported earlier [11]. The work presented here would be an ideal scenario for screens conducted in arrayed formats comprised of one hairpin per gene per well. Intuitively, this phenomenon would become more complex when evaluating pooled screening formats which measure relative hairpin depletions as their end point readouts. A single shRNA pool may contain up to 50,000 hairpins targeting multiple genes collectively, and with an underlying assumption of single hairpin integration per cell. The data is then deconvoluted using microarray hybridization or next gen sequencing techniques to identify hits [10]. However, there are no means to control for multiple different hairpins transducing a single cell, questioning the phenotypic outcome of being target specific or synergistic. This inherent noise and heterogeneity in pools when taken into account with the ATSG machinery per hairpin would produce multifold of targeting sequences inside a single cell. Therefore, the implications of ATSG in pooled screens would be far more widespread, introducing high ambiguity and misleading data interpretations heavily.

Our study has presented one aspect of the issues faced by RNAi in terms of targeting sequences; another aspect would be the targeted sequence itself. There is a growing awareness about the dynamic nature of the genome; individual cells can harbor high genetic variability [42], more so in the case of *in vitro* cell cultures. Recently, it has also been shown that the efficiency of RNAi is impacted by variations in levels of gene expression and physiological conditions [43]. Altogether, it is important to bear in mind that expected specificities of RNAi can get compromised at multiple levels, so deeper understanding of the technology is required to address these challenges aptly. Amidst this turmoil with RNAi, an alternate genome-editing technology called clustered regularly interspaced short palindromic repeats (CRISPR) has gathered active interest [44]–[45]. However, CRISPR also faces issues pertaining to its design and delivery into eukaryotic cells, cell-type dependencies as well as non-specific targeting [44]–[45]. Bearing in mind the lessons learnt from an early adoption of RNAi, it is critical to first optimally address the uses and limitations of CRISPR before embracing this technology as the ‘next big thing’.

In summary, with the discovery of ATSG, we have uncovered yet another facet of non-specific RNAi gene silencing in plasmid-based hairpins, extendable to miR30-based backbones. This dicer independent and cell-type dependent alternate processing pathway is emerging as a culprit behind misleading RNAi screening outcomes; especially across a multitude of cell lines. It is a matter of concern that a single shRNA hairpin is capable of generating up to six alternate interfering RNAs; thus, in a pooled set up, one would indeed expect a multiplicity of these interfering sequences across thousands and thousands of hairpins to result in a totally random chaotic gene knockdown process; supported by the ever increasing lack of gene target confirmation and reproducibility from pooled shRNA screens to date [4]. It is our hope that this study provides the strongest line of evidence yet in support of arrayed shRNA screening and begs the question as to the scientific merits of any pooled approach.

## Supporting Information

Table S1 Custom designed 78 siRNA duplexes to target potential OTEs of TRCN#40273. The matching nucleotides are color coded red, the remaining siRNA duplex strand is color coded blue.
(DOCX)
