# Supplementary material for: Discovery of a Dicer-Independent, Cell-Type Dependent Alternate Targeting Sequence Generator: Implications in Gene Silencing & Pooled RNAi Screens
Source: PLoS One. 2014 Jul 2;9(7):e100676. doi: 10.1371/journal.pone.0100676 (PMC4079264; doi:10.1371/journal.pone.0100676)
Supplement: Table S1 — Custom designed 78 siRNA duplexes to target potential OTEs of TRCN#40273. The matching nucleotides are color coded red, the remaining siRNA duplex strand is color coded blue. (DOCX) [file pone.0100676.s001.docx]

**Table S1.** Custom designed 78 siRNA duplexes to target potential OTEs of TRCN#40273.

| **Alternate Targets** | **Location on hairpin oligonucleotide** | **Cleavage start position** | **Match**  **length** | **Seed match** | **siRNA (guide)** |
| --- | --- | --- | --- | --- | --- |
| **CTTN ^1^** | CCGGCGGCAAATACGGTATCGACAACTCGAGTT**GTCGATACCGTATTTGCCG**TTTTTG | 33 | n/a | n/a | **GTCGATACCGTATTTGCCG** |
| ASH1L | CCGGCGGCA**AATACGGTATC**GACAACTCGAGTTGTCGATACCGTATTTGCCGTTTTTG | 10 | 11 | Yes | **AATACGGTATC**GACAACTC |
| ASH1L | CCGGCGGCAAATACGGTATCGACAACTCGAGTTGTC**GATACCGTATT**TGCCGTTTTTG | 29 | 11 | - | GAGTTGTC**GATACCGTATT** |
| BRWD3 | CCGGCGGCAAA**TACGGTATCGA**CAACTCGAGTTGTCGATACCGTATTTGCCGTTTTTG | 12 | 11 | Yes | **TACGGTATCGA**CAACTCGA |
| BRWD3 | CCGGCGGCAAATACGGTATCGACAACTCGAGTTG**TCGATACCGTA**TTTGCCGTTTTTG | 34 | 11 | Yes | G**TCGATACCGTA**TTTGCCG |
| C7ORF23 | CCGGCGGCAAATAC**GGTATCGACAA**CTCGAGTTGTCGATACCGTATTTGCCGTTTTTG | 15 | 11 | Yes | **GGTATCGACAA**CTCGAGTT |
| C7ORF23 | CCGGCGGCAAATACGGTATCGACAACTCGAG**TTGTCGATACC**GTATTTGCCGTTTTTG | 24 | 11 | - | AACTCGAG**TTGTCGATACC** |
| CD37 | CCGGCGGCAAAT**ACGGTATCGA**CAACTCGAGTTGTCGATACCGTATTTGCCGTTTTTG | 13 | 10 | Yes | **ACGGTATCGA**CAACTCGAG |
| CDK13 | CCGGCGGC**AAATACGGTAT**CGACAACTCGAGTTGTCGATACCGTATTTGCCGTTTTTG | 9 | 11 | Yes | **AAATACGGTAT**CGACAACT |
| CLDN15 | CCGGC**GGCAAATACGG**TATCGACAACTCGAGTTGTCGATACCGTATTTGCCGTTTTTG | 6 | 11 | Yes | **GGCAAATACGG**TATCGACA |
| CLDN15 | CCGGCGGCAAATACGGTATCGACAACTCGAGTTGTCGATA**CCGTATTTGCC**GTTTTTG | 33 | 11 | - | TGTCGATA**CCGTATTTGCC** |
| COL8A1 | CCGGCGGCA**AATACGGTAT**CGACAACTCGAGTTGTCGATACCGTATTTGCCGTTTTTG | 10 | 10 | Yes | **AATACGGTAT**CGACAACTC |
| DROSHA | CCGGCGGCAAATACGGTATCGACAACTCG**AGTTGTCGAT**ACCGTATTTGCCGTTTTTG | 30 | 10 | Yes | **AGTTGTCGAT**ACCGTATTT |
| EBF3 | CCGGCGGCAAATACG**GTATCGACAAC**TCGAGTTGTCGATACCGTATTTGCCGTTTTTG | 16 | 11 | Yes | **GTATCGACAAC**TCGAGTTG |
| EBF3 | CCGGCGGCAAATACGGTATCGACAACTCGA**GTTGTCGATAC**CGTATTTGCCGTTTTTG | 23 | 11 | - | CAACTCGA**GTTGTCGATAC** |
| EEFSEC | CCGGCGGC**AAATACGGTATC**GACAACTCGAGTTGTCGATACCGTATTTGCCGTTTTTG | 9 | 12 | Yes | **AAATACGGTATC**GACAACT |
| EEFSEC | CCGGCGGCAAATACGGTATCGACAACTCGAGTTGTC**GATACCGTATTT**GCCGTTTTTG | 37 | 12 | Yes | **GATACCGTATTT**GCCGTTT |
| FBN1 | CCGGCGGCAAATACGGTATCGA**CAACTCGAGTTG**TCGATACCGTATTTGCCGTTTTTG | 23 | 12 | Yes | **CAACTCGAGTTG**TCGATAC |
| FKTN | CCGGCGGCAAATAC**GGTATCGACAA**CTCGAGTTGTCGATACCGTATTTGCCGTTTTTG | 15 | 11 | Yes | **GGTATCGACAA**CTCGAGTT |
| FKTN | CCGGCGGCAAATACGGTATCGACAACTCGAG**TTGTCGATACC**GTATTTGCCGTTTTTG | 24 | 11 | - | AACTCGAG**TTGTCGATACC** |
| GGNBP2 | CCGGCGGCAAATACGGTATC**GACAACTCGAGT**TGTCGATACCGTATTTGCCGTTTTTG | 21 | 12 | Yes | **GACAACTCGAGT**TGTCGAT |
| GGNBP2 | CCGGCGGCAAATACGGTATCGACA**ACTCGAGTTGTC**GATACCGTATTTGCCGTTTTTG | 25 | 12 | Yes | **ACTCGAGTTGTC**GATACCG |
| GNG7 | CCGGCGGCAAATAC**GGTATCGACAACTC**GAGTTGTCGATACCGTATTTGCCGTTTTTG | 15 | 14 | Yes | **GGTATCGACAACTC**GAGTT |
| GNG7 | CCGGCGGCAAATACG**GTATCGACAACTC**GAGTTGTCGATACCGTATTTGCCGTTTTTG | 16 | 13 | Yes | **GTATCGACAACTC**GAGTTG |
| GNG7 | CCGGCGGCAAATACGG**TATCGACAACTC**GAGTTGTCGATACCGTATTTGCCGTTTTTG | 17 | 12 | Yes | **TATCGACAACTC**GAGTTGT |
| GNG7 | CCGGCGGCAAATACGGTATCGACAACTC**GAGTTGTCGATACC**GTATTTGCCGTTTTTG | 29 | 14 | Yes | **GAGTTGTCGATACC**GTATT |
| GPR137C | CCGGCGGCAAATACGGTATCG**ACAACT**C**GAGTTGT**CGATACCGTATTTGCCGTTTTTG | 22 | 14 | Yes | **ACAACT**C**GAGTTGT**CGATA |
| IDI2-AS1 | CCGGCGGCAAATACGGTATCGACAACTCGAGTTGTCG**ATACCGTATTT**GCCGTTTTTG | 38 | 11 | Yes | **ATACCGTATTT**GCCGTTTT |
| KLHL7 | CCGGCGGCAAATACGGTATCGACAACTCGA**GTTGTCGATAC**CGTATTTGCCGTTTTTG | 31 | 11 | Yes | **GTTGTCGATAC**CGTATTTG |
| KLHL7 | CCGGCGGCAAATACG**GTATCGACAAC**TCGAGTTGTCGATACCGTATTTGCCGTTTTTG | 8 | 11 | - | CAAATACG**GTATCGACAAC** |
| MTX3 | CCGGCGGCAAATACGGTATCGAC**AACTCGAGTTG**TCGATACCGTATTTGCCGTTTTTG | 24 | 11 | Yes | **AACTCGAGTTG**TCGATACC |
| MTX3 | CCGGCGGCAAATACGGTATCGA**CAACTCGAGTT**GTCGATACCGTATTTGCCGTTTTTG | 15 | 11 | - | GGTATCGA**CAACTCGAGTT** |
| MYO3B | CCGGCGGCAAATACGGTATC**GACAACTCGAG**TTGTCGATACCGTATTTGCCGTTTTTG | 21 | 11 | Yes | **GACAACTCGAG**TTGTCGAT |
| MYO3B | CCGGCGGCAAATACGGTATCGACAA**CTCGAGTTGTC**GATACCGTATTTGCCGTTTTTG | 18 | 11 | - | ATCGACAA**CTCGAGTTGTC** |
| NTN1 | CCGGCGGCAAATACGGTA**TCGACAACTCG**AGTTGTCGATACCGTATTTGCCGTTTTTG | 19 | 11 | Yes | **TCGACAACTCG**AGTTGTCG |
| NTN1 | CCGGCGGCAAATACGGTATCGACAACT**CGAGTTGTCGA**TACCGTATTTGCCGTTTTTG | 20 | 11 | - | CGACAACT**CGAGTTGTCGA** |
| PRKCH | CCGGCGGCAAATAC**GGTATCGACAACT**CGAGTTGTCGATACCGTATTTGCCGTTTTTG | 15 | 13 | Yes | **GGTATCGACAACT**CGAGTT |
| PRKCH | CCGGCGGCAAATACG**GTATCGACAACT**CGAGTTGTCGATACCGTATTTGCCGTTTTTG | 16 | 12 | Yes | **GTATCGACAACT**CGAGTTG |
| PRKCH | CCGGCGGCAAATACGGTATCGACAACTCG**AGTTGTCGATACC**GTATTTGCCGTTTTTG | 30 | 13 | Yes | **AGTTGTCGATACC**GTATTT |
| RBM33 | CCGGCGGCAAATACGGTATC**GACAACTCGAGTT**GTCGATACCGTATTTGCCGTTTTTG | 21 | 13 | Yes | **GACAACTCGAGTT**GTCGAT |
| RBM33 | CCGGCGGCAAATACGGTATCGAC**AACTCGAGTTGTC**GATACCGTATTTGCCGTTTTTG | 24 | 13 | Yes | **AACTCGAGTTGTC**GATACC |
| SCAND3 | CCGGCGGCAAATACGGTATCGACAACTCGAGTTGTCG**ATACCGTATTT**GCCGTTTTTG | 38 | 11 | Yes | **ATACCGTATTT**GCCGTTTT |
| SLC6A6 | CCGGCGGC**AAATACGGTAT**CGACAACTCGAGTTGTCGATACCGTATTTGCCGTTTTTG | 9 | 11 | Yes | **AAATACGGTAT**CGACAACT |
| ST8SIA1 | CCGGCGGCAAATACGGTATCG**ACAACTCGAGT**TGTCGATACCGTATTTGCCGTTTTTG | 22 | 11 | Yes | **ACAACTCGAGT**TGTCGATA |
| STATH | CCGGC**GGCAAATACG**GTATCGACAACTCGAGTTGTCGATACCGTATTTGCCGTTTTTG | 6 | 10 | Yes | **GGCAAATACG**GTATCGACA |
| STATH | CCGGCGGCAAATACGGTATCGACAACTCGAGTTGTCGATAC**CGTATTTGCC**GTTTTTG | 33 | 10 | - | TGTCGATAC**CGTATTTGCC** |
| THEM4 | CCGGCGGCAAATACGG**TATCGACAACTC**GAGTTGTCGATACCGTATTTGCCGTTTTTG | 17 | 12 | Yes | **TATCGACAACTC**GAGTTGT |
| THEM4 | CCGGCGGCAAATACGGTATCGACAACTC**GAGTTGTCGATA**CCGTATTTGCCGTTTTTG | 29 | 12 | Yes | **GAGTTGTCGATA**CCGTATT |
| ABRA | CCGGCGGCAAATACGGTATCGACAACTCGAGTTGTCG**ATACCGTATTT**GCCGTTTTTG | 35 | 11 | - | TCG**ATACCGTATTT**GCCGT |
| AIM1 | CCGGCGGCAAATACGGTATCGACAACTCGAGTTGTCGATACCGT**ATTTGCCGTT**TTTG | 36 | 10 | - | CGATACCGT**ATTTGCCGTT** |
| BRSK2 | CCGGCGGCAAATACGGTATCGACAACTCGAGTTGTCGATACCG**TATTTGCCGTTTT**TG | 40 | 13 | - | ACCG**TATTTGCCGTTTT**TG |
| CCT6B | CCGGCGGCAAATACGGTATCGACAACTCGAGTTGTCGATACCGTAT**TTGCCGTTTTTG** | 40 | 12 | - | ACCGTAT**TTGCCGTTTTTG** |
| DNAJC18 | CCGGCG**GCAAATACGGT**ATCGACAACTCGAGTTGTCGATACCGTATTTGCCGTTTTTG | 6 | 11 | Yes | G**GCAAATACGGT**ATCGACA |
| DNAJC18 | CCGGCGGCAAATACGGTATCGACAACTCGAGTTGTCGAT**ACCGTATTTGC**CGTTTTTG | 33 | 11 | - | TGTCGAT**ACCGTATTTGC**C |
| DOK6 | CCGGCGGCAAATACGGTATCGACAACTCGAGTTGTCGATACCGTA**TTTGCCGTTTTT**G | 39 | 12 | - | TACCGTA**TTTGCCGTTTTT** |
| DUS2L | CCGGCGGCAAATACGGTATC**GACAACTCGA**GTTGTCGATACCGTATTTGCCGTTTTTG | 20 | 10 | Yes | C**GACAACTCGA**GTTGTCGA |
| ENOSF1 | CCGGCGGCAAATAC**GGTATCGACA**ACTCGAGTTGTCGATACCGTATTTGCCGTTTTTG | 14 | 10 | Yes | C**GGTATCGACA**ACTCGAGT |
| ENOSF1 | CCGGCGGCAAATACGGTATCGACAACTCGAGT**TGTCGATACC**GTATTTGCCGTTTTTG | 25 | 10 | - | ACTCGAGT**TGTCGATACC**G |
| FRY | CCGGCGGCAAATAC**GGTATCGACA**ACTCGAGTTGTCGATACCGTATTTGCCGTTTTTG | 14 | 10 | Yes | C**GGTATCGACA**ACTCGAGT |
| FRY | CCGGCGGCAAATACGGTATCGACAACTCGAGT**TGTCGATACC**GTATTTGCCGTTTTTG | 25 | 10 | - | ACTCGAGT**TGTCGATACC**G |
| FSIP2 | CCGGCGGCAAATACGGTATCGACAACTCGA**GTTGTCGAT**A**CCGT**ATTTGCCGTTTTTG | 27 | 14 | - | TCGA**GTTGTCGATACCGT**A |
| GREM1 | CCGGCGGCAAATACGGTATCGACAACTCGAGTTGTCGATACCGTA**TTTGCCGTTTTT**G | 39 | 12 | - | TACCGTA**TTTGCCGTTTTT** |
| GRIA4 | CCGGCGGCAAATACGGTATCGACAACTCGAGTTGTCGATACC**GTATTTGCC**G**TTTTTG** | 40 | 16 | - | ACC**GTATTTGCC**G**TTTTTG** |
| HNRPDL | CCGGC**GGCAAATACG**GTATCGACAACTCGAGTTGTCGATACCGTATTTGCCGTTTTTG | 5 | 10 | Yes | C**GGCAAATACG**GTATCGAC |
| HNRPDL | CCGGCGGCAAATACGGTATCGACAACTCGAGTTGTCGATAC**CGTATTTGCC**GTTTTTG | 34 | 10 | - | GTCGATAC**CGTATTTGCC**G |
| KCNJ16 | CCGGCGGCAAATACGGTATCG**ACAACTCGAGT**TGTCGATACCGTATTTGCCGTTTTTG | 18 | 11 | - | ATCG**ACAACTCGAGT**TGTC |
| KCNJ16 | CCGGCGGCAAATACGGTATCGACA**ACTCGAGTTGT**CGATACCGTATTTGCCGTTTTTG | 21 | 11 | - | GACA**ACTCGAGTTGT**CGAT |
| MIR548AL | CCGGCGGCAAATACGGTATCGACAACTCGAGTTGTCGATACCGTAT**TTGCCGTTTTTG** | 40 | 12 | - | ACCGTAT**TTGCCGTTTTTG** |
| PEX13 | CCGGCGGCAAATACGGTATCGACAACTCGAGTTGTC**GATACCGTAT**TTGCCGTTTTTG | 36 | 10 | Yes | C**GATACCGTAT**TTGCCGTT |
| PGM3 | CCGGCGGCAAATACGGTATCGACAACTCGAGTTGTCGATACCGT**ATTTGCCGTTTT**TG | 40 | 12 | - | ACCGT**ATTTGCCGTTTT**TG |
| PHLDB2 | CCGGCGGCAAATACGGTATCGACAACTCGAGTTGTCGATACCGT**ATTTGCCGTTTT**TG | 39 | 12 | - | TACCGT**ATTTGCCGTTTT**T |
| PPP1R16B | CCGGCGGCAAATACGGT**ATCGACAACT**CGAGTTGTCGATACCGTATTTGCCGTTTTTG | 10 | 10 | - | AATACGGT**ATCGACAACT**C |
| RABGAP1L | CCGGCGGCAAATACGGTATCGACAACTCGAGTTGTCGATACCGTA**TTTGCCGTTTTT**G | 39 | 12 | - | TACCGTA**TTTGCCGTTTTT** |
| SEZ6 | CCGGCGGCAAATACGGTATCGACAACTCGAGTTGTCGA**TACCGTATTTG**CCGTTTTTG | 36 | 11 | - | CGA**TACCGTATTTG**CCGTT |
| SHISA9 | CCGGCGGCAAATACGGTATCGACAACTCGAGTTGTCGATACCGT**ATTTGCCGTTTTT**G | 39 | 13 | - | TACCGT**ATTTGCCGTTTTT** |
| ST7 | CCGGCGGCAAATACGGTATCGACAACTCGAGTTGTCGATA**CCGTATTTGC**CGTTTTTG | 32 | 10 | - | TTGTCGATA**CCGTATTTGC** |
| STK10 | CCGGCGGCAAATACGGTATCGACAACTCGAGTTGTCGATACCG**TATTTGCCGT**TTTTG | 35 | 10 | - | TCGATACCG**TATTTGCCGT** |
| WDR92 | CCGGCGGCAAATACGGTATCGACAACTCGAGTTGTCGATACCGTAT**TTGCCGTTTTTG** | 40 | 12 | - | ACCGTAT**TTGCCGTTTTTG** |

^1^ Target gene of hairpin TRC#40273

The matching nucleotides are color coded red, the remaining siRNA strand is color coded blue
